# Genetic analysis and clinical characteristics of sporadic and familial congenital cataracts in southern Chinese families

**DOI:** 10.3389/fgene.2026.1744173

**Published:** 2026-02-26

**Authors:** Teng Huang, Hai-Sen Sun, Ya-Nan Liu, Qiu-Ling Xie, Yang Liu, Xue-Chuan Miao, Wenhui Wu, Jin Li

**Affiliations:** 1 National Clinical Research Center for Ocular Diseases, Eye Hospital, Wenzhou Medical University, Wenzhou, China; 2 Cataract Center, The People’s Hospital of Hebi, Hebi, China; 3 Institute of PSI Genomics, Wenzhou Global Eye and Vision Innovation Center, Wenzhou, China

**Keywords:** congenital cataract, genetic variants, southern Chinese families, sporadic and familial cases, whole-exome sequencing

## Abstract

**Introduction:**

Congenital cataract is a major cause of blindness and severe visual impairment in children. It may occur as an isolated ocular abnormality or in combination with microcornea, microphthalmia, aniridia, or glaucoma. It can also be part of syndromic conditions. Whole-exome sequencing (WES) is now recognized as an appropriate first-line approach for genetic testing in patients with congenital cataract. In this study, we use WES to characterize the genotype spectrum in a pediatric cataract cohort from southern China.

**Methods:**

In this study, we aimed to clarify the genetic basis of congenital cataract in 40 families from southern China by WES. All candidate variants were confirmed by Sanger sequencing. After bioinformatic analysis, we prioritized rare or novel variants predicted to have moderate to damaging effects and assessed their segregation within each family.

**Results:**

In this cohort of 40 probands with congenital cataract, pathogenic/likely pathogenic variants were identified in 15 (37.5%) individuals, including 6 sporadic cases and 9 familial cases. The identified variants involved 12 genes (*CRYBB3*, *CRYBB2*, *CRYGS*, *CRYAA*, *GJA8*, *MIP*, *NHS*, *BCOR*, *COL11A1*, *PAX6*, *FTL*, and *FYCO1*). In total, 15 pathogenic/likely pathogenic variants were detected, of which 7 were novel. Among genotype-positive patients, seven presented with syndromic cataract, whereas eight had non-syndromic cataract.

**Discussion:**

This study performed WES in 40 probands with congenital cataracts from southern China and achieved a molecular diagnostic yield of 37.5%. Pathogenic/likely pathogenic variants were predominantly identified in crystallin genes, genes encoding lens membrane proteins, and genes implicated in syndromic forms of disease. Notably, a substantial proportion of apparently sporadic cases harbored variants suggestive of a *de novo* origin. These findings support the clinical utility of WES in clarifying the genetic basis of genetically heterogeneous congenital cataract. They also underscore the limitations of WES compared with whole-genome sequencing (WGS) and highlight the need for larger cohorts and functional validation of candidate variants.

## Background

Congenital cataract is defined as the opacity of the crystalline lens that is present at birth or arises within the first year of life (Mei et al., 2022). The overall prevalence of congenital cataract (CC) has been estimated at 4.24 per 10,000 individuals, which classifies it as a rare disease according to World Health Organization (WHO) criteria. Regional differences in congenital cataract prevalence have been reported worldwide. In Asia, the estimated prevalence is highest, at 7.43 per 10,000 individuals (Wu et al., 2016). Although congenital cataract is relatively rare, it is a major cause of blindness and severe visual impairment in children. It accounts for approximately 5.0%–20.0% of pediatric blindness worldwide (Gilbert and Foster). Early diagnosis is particularly important. Timely and appropriate interventions can contribute substantially to improving vision (Gao et al., 2022). Nearly half of inherited cataracts follow an autosomal dominant pattern (OMIM #604307) (Berry et al., 2020a). Autosomal recessive (OMIM #614691) and X-linked (OMIM #302350) forms are less common (Berry et al., 2020b). Because congenital cataract is not life-threatening and usually does not affect fertility, inherited forms tend to show relatively high penetrance. As a result, the pathogenic variants can be stably transmitted from generation to generation (Gao et al., 2022). Congenital cataracts can present as isolated lens opacity, which is the most common form and accounts for approximately two-thirds of all congenital cataract cases. They may also occur in conjunction with other ocular developmental anomalies, such as microphthalmia, microcornea, and iris abnormalities, or as part of broader genetic syndromes (Haargaard et al., 2004). Congenital cataracts can be classified in several complementary ways. Most classification schemes are based on the anatomical location within the lens, the morphological pattern of the opacity, and the underlying etiology (Reddy et al., 2004; Hejtmancik, 2008; Amaya et al., 2003). According to the location and shape of the lens opacity, congenital cataracts can be divided into seven clinical types. These include nuclear cataract, polar cataract, lamellar cataract, nuclear with cortical cataract, cortical cataract, sutural cataract, and total cataract (Zhai et al., 2017). Different types of congenital cataracts lead to varying degrees of visual impairment in affected patients (Table 1).

**TABLE 1:** Genes associated with congenital cataracts.

Gene category	Related gene
Crystallin gene	*CRYAA*, *CRYAB*, *CRYBB1*, *CRYBB2*, *CRYBB3*, *CRYBA1/A3*, *CRYBA2*, *CRYBA4*, *CRYGC*, *CRYGD*, *CRYGS*
Membrane protein gene	*GJA3*, *GJA8*, *MIP*, *LIM2*
Growth and transcription factor gene	*PAX6*, *PITX3*, *MAF*, *HSF4*
Cytoskeletal proteins gene	*BFSP1*, *BFSP2*, *VIM*
X-linked syndromic cataract gene	*NHS*, *BCOR*, *OCRL*
L-Ferritin gene	*FTL*
Other genes	*FYCO1*, *COL11A1*

Next-generation sequencing (NGS) has substantially advanced the molecular diagnosis of genetically heterogeneous disorders. Whole-exome sequencing (WES) interrogates protein-coding regions and canonical splice sites, enabling efficient detection of disease-associated variants with lower sequencing requirements than whole-genome sequencing (WGS) (Majewski et al., 2011; Petersen et al., 2017). Accordingly, WES has become a widely used approach for genetic evaluation in clinically heterogeneous conditions, including congenital cataracts. Based on the latest update of the Cat-Map database (https://cat-map.wustl.edu/, last updated February 2025, accessed 28 December 2025) (Shiels, 2024), sequence variants in more than 500 genes have been associated with congenital cataracts. Notably, over 300 of these genes are related to syndromic congenital cataracts. Causative genes for congenital cataracts can be broadly categorized into several functional groups, including crystallins, lens membrane proteins, growth and transcription factors, cytoskeletal components, X-linked syndromic cataract genes, and other cataract-associated genes (Anand et al., 2018; Song et al., 2009). A consolidated overview of these gene categories and representative genes is provided in Table 1. Congenital cataracts show high genetic heterogeneity and phenotypic diversity. In this study, we performed whole-exome sequencing on 40 probands with bilateral congenital cataracts. Our objectives were to identify pathogenic or likely pathogenic variants, explore genotype–phenotype correlations, and expand the knowledge of clinically relevant mutations. The results are expected to enhance diagnostic accuracy, guide personalized management, and contribute to precision medicine in pediatric ophthalmology (Rechsteiner et al., 2021; Yu et al., 2021).

Pathogenic or likely pathogenic variants were identified in 15 of the 40 probands. These variants were distributed across 12 genes associated with congenital cataracts. The affected genes included *CRYAA*, *CRYBB2*, *CRYBB3*, and *CRYGS* (crystallin genes); *GJA8* and *MIP* (membrane protein genes); *PAX6* (a growth and transcription factor gene); *BCOR* and *NHS* (X-linked syndromic cataract genes); and *FTL*, *FYCO1*, and *COL11A1* (other associated genes). Among the 15 variants, 7 were novel. These findings expand the known mutational spectrum of congenital cataract and support the development of precision medicine in pediatric ophthalmology.

## Methods

### Ethical considerations and participant recruitment

This study was approved by the Research Ethics Committee of Wenzhou Medical University Laboratory (approval number: 2021–239-k-209) and adhered to the tenets of the Declaration of Helsinki. A total of 40 individuals with clinically confirmed bilateral congenital cataracts were recruited from the Pediatric Cataract Center of Wenzhou Medical University Eye Hospital (Wenzhou, China). Written informed consent was obtained from all adult participants and from the parents or legal guardians of minors prior to enrollment. All data were handled in a de-identified manner. Participants were assigned study-specific codes, and no personally identifiable information was included in the manuscript or Supplementary Material. Pedigrees presented in the Supplementary Material are anonymized and do not contain information sufficient to identify individual participants. Detailed family histories and medical records were carefully collected. The presence and type of cataract phenotype in both affected and unaffected individuals were confirmed by slit-lamp biomicroscopy. Patients with a history of intrauterine infection, drug exposure, metabolic disorders, or malnutrition were excluded. For genomic DNA analysis, a 2 mL sample of peripheral venous blood or oral mucosal tissue was collected. Genomic DNA was extracted using either the QIAGEN Blood DNA Kit (QIAGEN, Germany) or the Invitrogen™ MagMAX™ DNA Multi-Sample Ultra 2.0 Kit (Thermo Fisher Scientific, Norway), following the manufacturer’s instructions. All probands presented with bilateral congenital cataracts, identified at birth or diagnosed within the first year of life, with timing supported by medical records, a consistent parental report of onset, or both. Some patients also showed microcornea and other ocular features, including microphthalmia, nystagmus, and glaucoma. In addition, a few patients exhibited extraocular manifestations, such as dental dysmorphologies, proteinuria, micrognathia, and polycystic kidney disease (Supplementary Table S1).

### Library preparation and next-generation sequencing

For genetic analysis, genomic DNA from affected individuals underwent WES. Library preparation was performed using the Twist Human Core Exome Kit (Twist Bioscience, United States), and sequencing was carried out on the NovaSeq 6000 platform (Illumina, San Diego, United States). Sequence reads were aligned to the human reference genome (hg19/GRCh37). The protocols for next-generation sequencing and downstream data analysis, including copy-number variation analysis, were described previously (Huang et al., 2017). In summary, variants were filtered to retain only novel variants that were absent from the public control databases Kaviar (https://db.systemsbiology.net/kaviar/) and the Genome Aggregation Database (gnomAD v4.1.0, http://gnomad.broadinstitute.org). In addition, rare variants with a gnomAD allele frequency <0.0001 were kept.

### Bioinformatics analysis

Variants were scrutinized for potential pathogenic clinical significance based on the Association for Clinical Genomic Science (ACGS) Best Practice Guidelines for Variant Classification in Rare Disease 2024 (v1.2) (Durkie et al., 2024) and the ClinGen Sequence Variant Interpretation (SVI) Group’s recommendations (Abou Tayoun et al., 2018; Ghosh et al., 2018). This analysis was based on a comprehensive review of previous literature reports, along with computational, functional, and population data. Confirmed variants underwent annotation using ANNOVAR (http://wannovar.wglab.org/), and respective minor allele frequencies were assessed in dbSNP (http://www.ncbi.nlm.nih.gov/projects/SNP), 1000 Genomes (http://www.1000genomes.org/), Exome Aggregation Consortium (ExAC) databases (http://exac.broadinstitute.org/), gnomAD (gnomAD v4.1.0, http://gnomad.broadinstitut.e.org/), and the PSI Gene Chinese-specific database. Additionally, prediction algorithms such as PolyPhen-2 (Adzhubei et al., 2010) (version 2.2.2, 2012, http://genetics.bwh.harvard.edu/pph2/), MutationTaster (Schwarz et al., 2010) (version 2, 2012, http://www.mutationtaster.org/), MutationAssessor (Reva et al., 2011) (http://mutationassessor.org), SpliceAI (de Sainte Agathe et al., 2023), REVEL (Ioannidis et al., 2016), and CADD (Rentzsch et al., 2019) (http://cadd.gs.washington.edu) and disease and phenotype databases including Online Mendelian Inheritance in Man (OMIM; http://www.omim.org), ClinVar (http://www.ncbi.nlm.nih.gov/clinvar), the Human Gene Mutation Database (HGMD; http://www.hgmd.org), and Human Phenotype Ontology (HPO; https://hpo.jax.org/app/) were used for variant annotation and interpretation. The above database was accessed by February 2024.

Multiple protein sequence alignments were conducted using T-COFFEE and Jalview (Waterhouse et al., 2009) to assess cross-species conservation. Online resources such as UniProt (https://www.uniprot.org) and SMART (smart.embl-heidelberg.de) were utilized to analyze alterations in protein properties for assessing secondary structure. Protein and nucleotide sequences were visualized using IBS (Illustrator for Biological Sequences). Three-dimensional (3D) models of both wild-type and mutant proteins were generated using the SWISS-MODEL server program. PyMOL was used to prepare illustrations (Supplementary Figure S1).

### Sanger sequencing

Validation of candidate variants by Sanger sequencing was performed in all probands and available family members. Primers were designed to amplify the specific DNA fragments of interest, and polymerase chain reaction (PCR) was carried out under standard conditions. The PCR products were then sequenced on an ABI 3730xl DNA Analyzer (Applied Biosystems, United States). The resulting sequences were compared with the corresponding reference sequences using MutationMapper software.

## Results

### Sequencing coverage metrics (captured regions)

WES was performed on genomic DNA from 40 probands with congenital cataracts to detect disease-associated variants. In total, 12.36 billion bases were generated, with an average of 73.25 million reads per chip. This provided approximately 98.2% coverage of the targeted regions and an average sequencing depth of 93.26× for each sample (Supplementary Table S3).

### Identification of suspected causative variants

**FIGURE 1:**
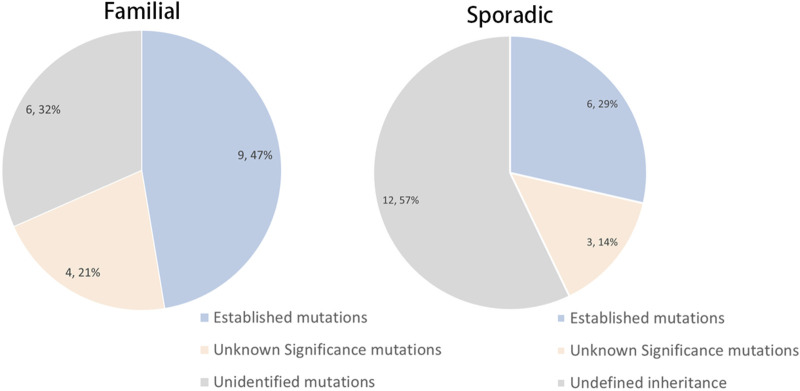
Mutation spectrum of familial and sporadic pediatric cataract cases. The mutation detection rates in the familial and sporadic cases were 47.4% and 28.6%, respectively.

**FIGURE 2:**
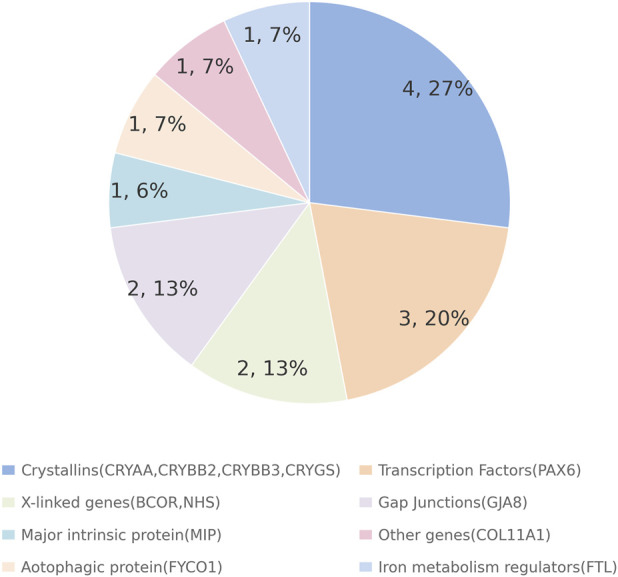
Distribution of mutations identified in known and novel candidate genes for cataract by NGS. Mutations were found in 12 different genes in sporadic and familial congenital cataract cases. These mutations occurred in genes encoding crystallins, X-linked syndromic proteins, transcription factors, gap junctions, major intrinsic proteins, and other proteins (FYCO1 and FTL). The relative proportions are illustrated in this diagram.

Overall, 19 of the 40 cases (47.5%) were familial. Pedigree analysis of these 19 families indicated autosomal dominant inheritance in 18 families, while the remaining family exhibited an X-linked dominant pattern. Pathogenic or likely pathogenic variants were identified in 15 of the 40 probands (Table 2). The variant detection yields were 47.4% (9/19) in familial cases and 28.6% (6/21) in sporadic cases (Figure 1). These pathogenic/likely pathogenic variants were distributed across 12 genes previously implicated in congenital cataract. Variants in crystallin genes (*CRYAA*, *CRYBB2*, *CRYBB3*, and *CRYGS*) were observed in 10.0% (4/40) of the cohort. Notably, variants were detected in PAX6 in three families and GJA8 in two families. In addition, single families harbored variants in BCOR, FTL, FYCO1, MIP, NHS, and COL11A1 (Figure 2). Overall, 15 pathogenic/likely pathogenic variants were identified, including 7 novel variants and 8 previously reported variants. Variant classification was performed in accordance with the ACGS guidelines for sequence variant interpretation; all variants were classified as pathogenic or likely pathogenic. Most familial cases were associated with autosomal dominant mutations in crystallin genes, except for one family carrying an X-linked NHS mutation. Sporadic cases were mainly explained by autosomal dominant mutations in a broader set of genes, including crystallin, gap junction, and transcription factor genes. In addition, *de novo* mutations in the X-linked gene *BCOR* were detected in unrelated families, and one autosomal recessive case was associated with a *FYCO1* mutation. Seven variants were classified as “variants of uncertain significance” and were identified in four familial and three sporadic cases (Supplementary Table S3; Supplementary Figure S2). The remaining six familial cases and fourteen sporadic patients with congenital cataracts had no variants of interest detected in this analysis (Supplementary Figure S3).

**TABLE 2:** Familial/sporadic bilateral congenital cataracts with pathogenic or likely pathogenic variants.

Family	Sex	Inheritance, before/After testing	Gene (refseq ID)	OMIM ID	Nucleotide change (Zygosity)	Predicted amino acid change	Ocular phenotype	SpliceAI	CADD	PolyPhen/MutationTaster/MutationAssessor	REVEL	ACGS	Novel
F#1	F	AD	CRYBB3 (NM_004076.5)	123,630	c.466G>A (het)	p.Gly156Arg	Lamellar	-	26.6	D,D,H	0.958	LP	Ref (Jackson et al., 2020)
F#2	F	AD	CRYBB2 (NM_000496.3)	123,620	c.562C>T (het)	p.Arg188Cys	N/K	-	23.5	D,D,H	0.790	P	Ref (Rechsteiner et al., 2021)
F#3	F	AD	CRYGS (NM_017541.4)	123,730	c.248G>A (het)	p.Cys83Tyr	Embryonic nuclear	-	33	D,D,H	0.748	LP	No
S#1	M	Sporadic/new AD	CRYAA (NM_000394.4)	123,580	c.34C>T (het)	p.Arg12Cys	Embryonic nuclear	-	28.5	D,D,M	0.901	P	Ref (Nallanthighal et al., 2021)
S#2	M	Sporadic/new AD	GJA8 (NM_005267.5)	600,897	c.133T>A (het)	p.Trp45Arg	N/K	-	27.5	D,D,M	0.952	P	Ref (Hansen et al., 2007)
F#4	F	AD	GJA8 (NM_005267.5)	600,897	c.131T>C (het)	p.Val44Ala	Embryonic nuclear	-	26.1	D,D,L	0.974	P	Ref (Li et al., 2016)
F#5	F	AD	MIP (NM_012064.4)	154,050	c.657C>A (het)	p.Tyr219*	Lamellar	-	35	-,D,-	-	LP	Yes
F#6	M	XLD	NHS (NM_001291867.2)	300,457	c.766dup (hem)	p.Leu256Profs*21	Lamellar	-	-	-	-	P	Yes
S#3	F	Sporadic/XLD	BCOR (NM_001123385.2)	300,485	c.4862del (hem)	p.Pro1621fs	Posterior polar	-	-	-	-	P	Yes
S#7	M	AD/AR	COL11A1 (NM_001854.4)	120,280	c.3114 + 1G>A (het)	p.?	Cortical Cataract	1	33	_,D,_	-	LP	No
F#7	M	AD	PAX6 (NM_001368894.2)	607,108	c.400-1G>A (het)	p.?	Embryonic nuclear	0.9938	33	-,D,-	-	LP	Yes
F#8	F	AD	PAX6 (NM_001368894.2)	607,108	c.542dupC (het)	p.V182Gfs*32	Total	-	-	-	-	LP	Yes
S#5	M	Sporadic/likely new AD	PAX6 ELP4	607,108 606,985	CNV(deletion)	144.7 kb	Embryonic nuclear	-	-	-	-	P	Yes
F#9	F	AD	FTL (NM_000146.4)	134,790	c.-159G>C (het)	p.?	Coralliform cataract	-	-	-	-	LP	Ref ^[59]^
S#6	F	Sporadic/AR	FYCO1 (NM_024513.3) (NM_024513.4)	607,182	c.3588–9T>A c.2345_2346del (compound het)	p.Gln782fs	R Posterior polar L total	0.9214 -	15.22 -	-	- -	VUS P	Yes Yes

Abbreviations: Proband ID: F, family; S, sporadic; sex: F, female; M, male; D, damaging, P, possibly damaging; H, high; M, medium; L, low.

ACGS, Association for Clinical Genomic Science; P, pathogenic; LP, likely pathogenic; VUS, variant of unknown significance; Ref, reference.

### Variants in crystallin genes

**FIGURE 3:**
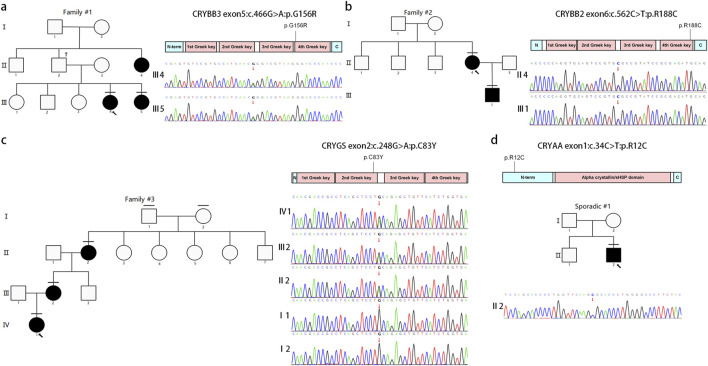
Pedigree and variants identified in crystallin genes. These schematics show the encoded domain structure of CRYBB3 **(a)**, CRYBB2 **(b)**, CRYGS **(c)**, and CRYAA **(d)**. Mutations found in this study are illustrated above the schematics. Squares and circles symbolize male and female individuals, respectively. Open and filled symbols indicate unaffected and affected individuals, respectively. The proband is marked with an arrow, and the horizontal line indicates individuals included in this study.

Variants in the crystallin genes were the most frequent mutations identified in this study (Zhuang et al., 2019). Pathogenic/likely pathogenic variants were detected in four probands: three familial and one sporadic case (Table 2). All familial cases were consistent with autosomal dominant inheritance, whereas the sporadic case was most consistent with a *de novo* autosomal dominant variant. All variants identified in these cases were missense mutations (Figure 3). The majority of variants localized to the Greek key motifs of crystallin proteins, which are essential for correct protein folding and the maintenance of lens transparency (Vendra et al., 2013).

### Variants in gap junction protein (*GJA8*) and major intrinsic protein (*MIP*)

Two probands in our cohort carried pathogenic variants in the gap junction gene *GJA8* (Table 2). One of these cases was sporadic and harbored a *de novo* heterozygous missense variant in exon 2 of *GJA8* (OMIM 600897), NM_005267.5:c.133T>A, p. (Trp45Arg). The proband presented with esotropia, nystagmus, and posterior staphyloma (Supplementary Table S1). Although a different nucleotide change at the same position in *GJA8* (OMIM 600897), NM_005267.5:c.133T>C, p. (Trp45Arg) has been reported previously, the affected individuals in that family showed a distinct phenotype. In addition to congenital cataracts, they presented with microcornea, microphthalmia, and a posterior capsule defect, indicating marked clinical heterogeneity compared with our proband (Zhang H. et al., 2019).

**FIGURE 4:**
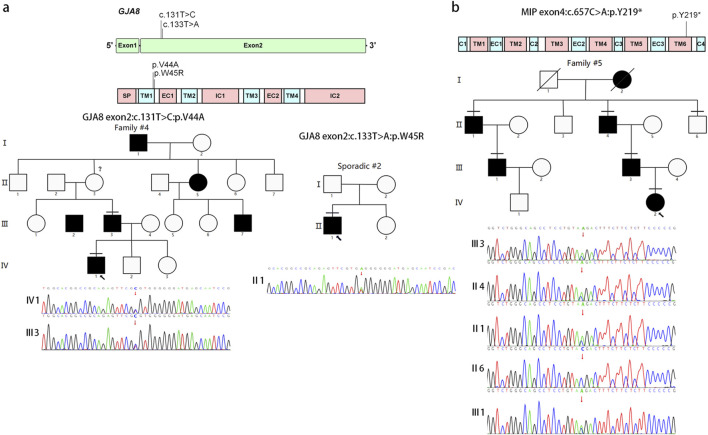
Pedigree and variants in GJA8 and MIP. These schematics show the exonic and encoded domain structure of GJA8 **(a)** and the encoded protein domain structure of MIP **(b)**. Mutations found in this study are illustrated above the schematics. The diagonal line indicates a deceased family member. The proband is marked with an arrow, and the horizontal line indicates individuals included in this study.

Another familial case carried a missense variant in *GJA8* (OMIM 600897), NM_005267.5:c.131T>C, p. (Val44Ala). In addition to congenital cataracts, the proband also presented with intermittent exotropia (Supplementary Table S1). This variant has been reported previously and functionally validated in *in vitro* cell-based assays (Zhu et al., 2014). The missense variants in the gap junction gene *GJA8* (OMIM 600897; NM_005267.5:c.131T>C, p. (Val44Ala); NM_005267.5:c.133T>A, p. (Trp45Arg)) are located in extracellular loop 1, close to the TM1/EC1 boundary (Figure 4a). These substitutions are predicted to selectively disrupt hemichannel gating while having less effect on fully formed gap junction channels. Dysfunctional hemichannels have been shown to contribute to the development of human congenital cataracts (Zhu et al., 2014; Beyer et al., 2013).

A novel nonsense variant in *MIP* (OMIM 154050), NM_012064.4:c.657C>A, p. (Tyr219Ter), was identified in familial case #5. This pedigree spanned four generations and included nine individuals, comprising six affected and three unaffected members; one affected individual was deceased. Clinical examination of all available family members revealed isolated lamellar cataracts in all affected individuals (Supplementary Table S1). Aquaporin 0 (AQP0), also known as the major intrinsic protein of the lens, is encoded by the MIP gene. The NM_012064.4:c.657C>A variant changes a highly conserved tyrosine codon (TAC) to a stop codon (TAA) at amino acid position 219 of AQP0, p. (Tyr219Ter) (Figure 4b). This nonsense change removes the entire intracellular C-terminal domain and produces a prematurely truncated protein. As a consequence, AQP0 is predicted to lose its function as a water channel in the cell membrane, which may lead to a congenital cataract phenotype (Song et al., 2015).

### Variants in X-Linked syndromic cataract genes *NHS and BCOR*

**FIGURE 5:**
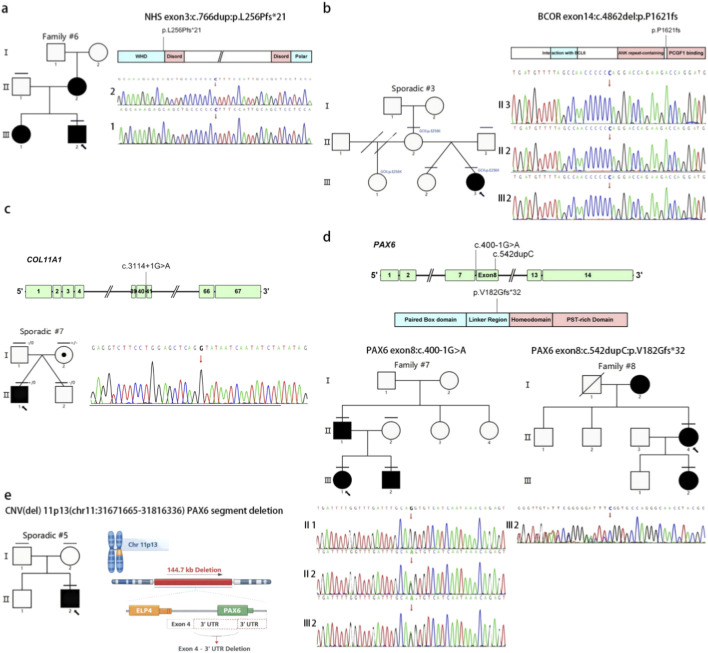
Pedigree and variants in NHS, BCOR, COL11A1, and PAX6. These schematics show the encoded domain structure of NHS **(a)**, BCOR **(b)**, COL11A1 **(c)**, and PAX6 **(d)**. NHS and BCOR are X-linked genes. These schematics show the encoded protein domain structure of NHS **(a)**, BCOR **(b)**, and COL11A1 **(c)** and the exonic and protein domain structure of PAX6 **(d)**. The variants found in this study are illustrated above the schematics. The diagonal line indicates a deceased family member. Double diagonal indicates divorce. The proband is marked with an arrow, and the horizontal line indicates individuals included in this study. A dotted circle indicates an obligate X-linked carrier. **(e)** Partial deletion of PAX6 (144.7 kb) at 11p13 identified in Sporadic #5.

X-linked syndromic cataracts were identified in two of the fifteen families. A hemizygous frameshift variant in the *NHS* (OMIM 300457), NM_001291867.2:c.766dup, p. (Leu256Profs*21), was identified in an individual from another family case #6 (Figure 5a). This variant has been reported previously (Chen et al., 2021). This frameshift mutation is predicted to result in a truncated protein. NHS is associated with X-linked Nance–Horan (Burdon et al., 2003) syndrome (Kammoun et al., 2018). Nevertheless, the proband presented only with bilateral cataracts and nystagmus, without clinical evidence of microphthalmia, dental anomalies, or other characteristic craniofacial features. This apparently incomplete phenotypic spectrum may reflect age-dependent penetrance, delayed manifestation of associated features, or both, given the proband’s young age (Supplementary Table S1).

In sporadic case #3, a *de novo* frameshift mutation, *BCOR* (OMIM 300485), NM_001123385:c.4862del, p. (Pro1621Argfs*53), was identified (Table 2). This mutation is responsible for X-linked oculo-facio-cardio-dental (OFCD) syndrome (Ng et al., 2004; Fan et al., 2009). The female proband (III:3) presented with bilateral posterior polar cataract, lower eyelid inversion, a broad nasal tip, and a patent foramen ovale (PFO). Dental anomalies included dysmorphologies of the teeth, delayed eruption, and features consistent with OFCD syndrome. Neither her parents nor her fraternal twin sister, who was also conceived via *in vitro* fertilization, tested positive for this mutation. The *BCOR* NM_001123385:c.4862del, p. (Pro1621Argfs*53) mutation is predicted to delete the entire PCGF1-binding domain. This domain is essential for interaction with PCGF1, a component of the polycomb group (PcG) multiprotein *BCOR* complex. This interaction is required to maintain the transcriptionally repressive state of BCL6 and CDKN1A (Figure 5b) (Junco et al., 2013). Additionally, the proband inherited maturity-onset diabetes of the young type 2 (MODY2) from her mother, associated with the *GCK* (OMIM 138079), NM_000162.5:c.766G>A, p. (Glu256Lys) mutation (Emelyanov et al., 2017).

### Variants in transcription factor gene *PAX6* in three families with complex cataract phenotypes

In *PAX6*, two heterozygous variants were detected. A familial splice mutation (OMIM 607108), NM_001368894.2:c.400-1G>A was found in Family #7, and a novel frameshift mutation (OMIM 607108), NM_001368894.2:c.542dup, p. (Val182Glyfs*32) was identified in Family #8 (Figure 5d). Furthermore, a sporadic case (#5) revealed a deletion of approximately 144.7 kb at chromosome 11p13. This region contains two RefSeq protein-coding genes, *ELP4* and *PAX6*, and was classified as “pathogenic” by ACGS. The variant caused a partial deletion of intron 4 and the 3′ UTR of the *PAX6* gene. qPCR validation confirmed that the proband was heterozygous, while his parents remained unaffected (Figure 5e). As a critical transcription factor, mutations in *PAX6* have the potential to affect various structures during development. *PAX6* mutations are characterized by the partial or complete absence of the iris, often accompanied by other ocular abnormalities such as cataracts and glaucoma (Zhang et al., 2011), corneal degeneration and microphthalmia (Lin et al., 2011), optic-nerve malformations (Azuma et al., 2003), and foveal hypoplasia and nystagmus (Khan and Aldahmesh, 2008). *PAX6* truncations are widely believed to be associated with aniridia, primarily due to haploinsufficiency (Dubey et al., 2015).

In Family #7, the proband (III:1) was referred with an embryonic nuclear cataract located nasally, accompanied by complete aniridia, nystagmus, and foveal hypoplasia. His affected father (II:1) and brother (III:2) harbored the same PAX6 variant and exhibited a comparable clinical phenotype. In Family #8, both the proband and her mother presented with complete aniridia, congenital cataract, and nystagmus. In contrast, the proband’s daughter currently manifests complete aniridia without evidence of congenital cataract, which may reflect age-dependent expressivity given her young age (9 months) (Figure 5d). Sporadic case #5 also presented with clinical symptoms of embryonic nuclear cataract, complete absence of the iris, nystagmus, and foveal hypoplasia, which are attributed to a partial deletion of PAX6. This PAX6 mutation accounts for his complex phenotype and may explain the suboptimal outcome following his cataract surgery (Supplementary Table S1) (Ma et al., 2016).

### Variants in the iron metabolism regulator (*FTL*), autophagic protein (*FYCO1*), and type XI collagen (*COL11A1*)

**FIGURE 6:**
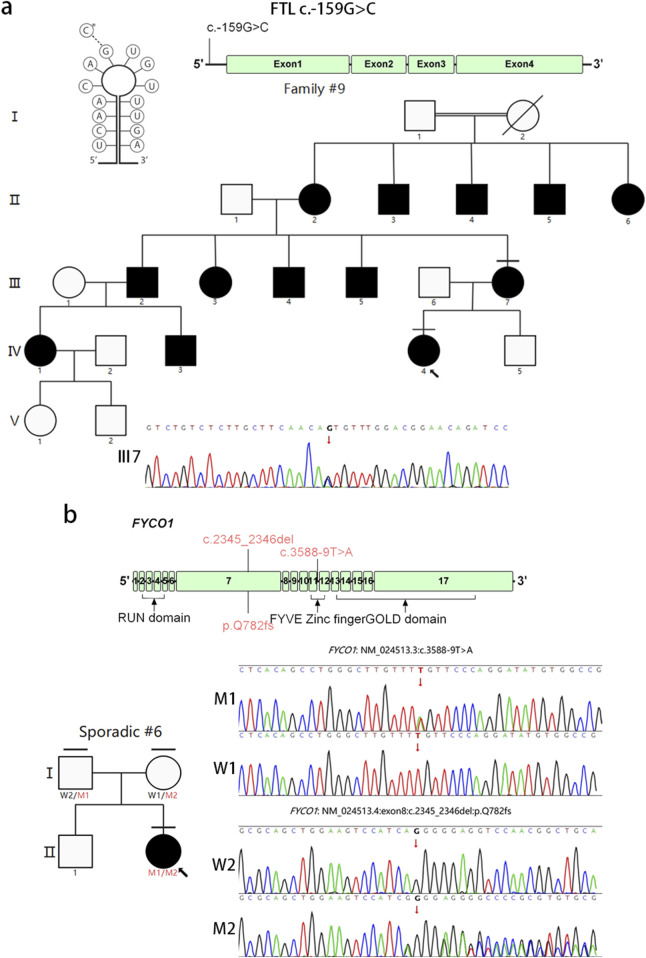
Pedigree and variants in FTL and FYCO1. These schematics show the exonic structure of FTL **(a)** and the exonic and protein domain structure of FYCO1 **(b)**. Mutations found in this study are illustrated above the schematics. The diagonal line indicates a deceased family member. The proband is marked with an arrow, and the horizontal line indicates individuals included in this study. W1, wild type 1; W2, wild type 2; M1, mutant type 1; M2, mutant type 2.

Family #9 consisted of 16 members across four generations. The proband (IV:4) was an 11-year-old female who presented with bilateral congenital coralliform cataracts. Her mother displayed a similar phenotype at an early age. Both the proband and her mother had a serum ferritin level of 2000.0 ng/ml. A previously reported mutation in *FTL* (OMIM 134790), NM_000146.4:c.-159G>C, known as the “Verona mutation” (Girelli et al., 1995), was found in both the proband and her mother. The affected individuals showed a heterozygous G>C change at position 41 from the transcription start site, within the third residue of the 5-base sequence (CAGUG) that characterizes the loop structure of the IRE. This *FTL* variant could be responsible for hereditary hyperferritinemia cataract syndrome (HHCS) (Meneses et al., 2011; Luscieti et al., 2013) (Figure 6a).

Novel compound heterozygous variants *FYCO1* (OMIM 607182), NM_024513.3:c.3588–9T>A, and *FYCO1* (OMIM 607182), NM_024513.4:c.2345_2346del, p. (Gln782Argfs?)* were identified in sporadic case #6. Parental segregation was confirmed (Figure 6b). The frameshift mutation c.2345_2346del, p. (Gln782Argfs?)* is predicted to truncate most of the coiled-coil region and result in the complete loss of the FYVE zinc-finger and GOLD domain. Additionally, the T-to-A transversion located at the conserved intron 11 donor splice site (c.3587 + 1G>T) may affect splicing (Li et al., 2018). All these variants are predicted to cause nonsense-mediated decay of the *FYCO1* mRNA, leading to a loss of *FYCO1* function (Chen et al., 2011). This loss occurs despite the need for turnover of large amounts of proteins and organelles during fiber cell differentiation (Chen et al., 2017).

In sporadic case #7, a splice variant in the *COL11A1* gene (OMIM 120280), NM_001854.4:c.3114 + 1G>A was identified. This mutation has been classified as likely pathogenic (LP) by the ACGS. The proband presented with bilateral congenital cortical cataracts. The right eye exhibited lens opacity covering approximately one-third of the pupil area, while the left eye showed opacity affecting about two-fifths of the pupil area. Both parents were unaffected and did not carry the mutation (Figure 5c). The *COL11A1* gene encodes type XI collagen, which is primarily expressed in cartilage, the lens of the eye, the cochlea, and other connective tissues (Nallanthighal et al., 2021). Mutations in this gene can lead to various clinical manifestations, particularly affecting the eyes, hearing, and skeletal system (Snead and Yates, 1999). Ocular manifestations of *COL11A1* mutations primarily involve lens opacity and glaucoma (Wang et al., 2023). In the ocular system, type XI collagen plays a crucial role in maintaining the structural integrity and transparency of the lens. Mutations typically disrupt collagen synthesis or assembly, resulting in abnormal optical properties of the lens (Boothe et al., 2020). Consequently, this leads to lens opacity and, in some cases, can result in vision impairment or blindness.

## Discussion

**FIGURE 7:**
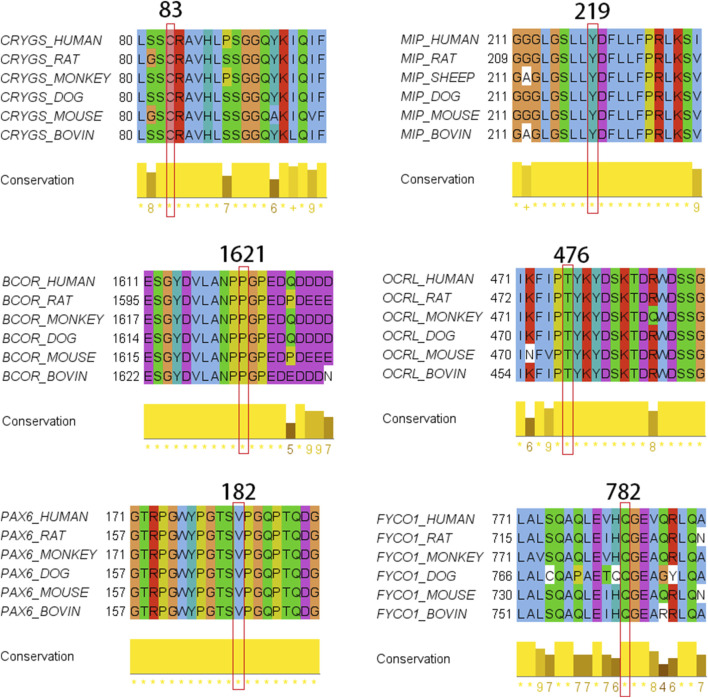
Multiple-sequence alignments from different vertebrate species.

We applied NGS to investigate the genetic etiology of congenital cataract in a cohort of 40 probands from southern China. Putative pathogenic variants were identified in 15 probands, involving 12 genes previously associated with congenital cataracts. The variant spectrum comprised missense, nonsense, frameshift, and splice-site changes. Notably, all missense variants mapped to functionally important protein domains. Frameshift deletions and nonsense variants were predicted to introduce premature termination codons, elicit nonsense-mediated mRNA decay, or both consistent with loss-of-function mechanisms. Moreover, all novel pathogenic variants identified in our families affected residues that are evolutionarily conserved across species (Figure 7). The variant *PAX6* (OMIM 607108), NM_001368894.2:c.400-1G>A, is a classical splicing variant, associated with loss-of-function (LOF) disease. The transcript containing this variant is biologically significant. It is expected to induce nonsense-mediated mRNA decay (NMD), which would impair the function of the protein encoded by the gene. SpliceAI predicts a score of ≥0.5 for this variant. The *FTL* (OMIM 134790), NM_000146.4:c.-159G>C variant is located within the iron response element (IRE) in the 5′-UTR of *FTL*. Mutations in the IRE of L-ferritin lead to constitutive, iron-independent ferritin expression (Cazzola and Skoda, 2000), which causes hereditary hyperferritinemia cataract syndrome (HHCS). The majority of these mutations are autosomal dominant (11/15), with autosomal recessive (1/15) and X-linked changes (2/15) also detected. The most frequently implicated genes were those encoding crystallins, which collectively accounted for 27.0% of the cohort. Several novel variants were identified across multiple gene categories, including crystallin genes and transcription factor genes, as well as less frequently reported congenital cataract-associated genes such as MIP and FYCO1, and the syndrome-associated gene BCOR. Interestingly, the two X-linked variants identified in our cohort were detected in dizygotic twins conceived via *in vitro* fertilization (IVF). Sanger sequencing confirmed that the unaffected siblings in both sibships did not harbor the corresponding variants. Collectively, these findings support a *de novo* origin of the variants in the affected twins. Our integrated approach, combining next-generation sequencing with familial segregation analysis, represents a significant advance in genetic diagnosis. It provides precise information for recurrence risk counseling and helps uncover clinically subtle or unrecognized syndromic associations.

Congenital cataract is a leading cause of treatable childhood blindness worldwide. It impairs vision by blocking or scattering light before it reaches the retina. This disruption occurs during the critical period of visual development and can result in irreversible visual loss if not treated promptly (Kandaswamy et al., 2020). Among the 15 causative variants identified in this study, 7 were novel: 4 detected in familial cases and 3 in apparently sporadic cases. The large number of genes associated with congenital cataracts, together with limited genotype–phenotype correlations, complicates clinical testing using traditional sequencing technologies. These challenges are particularly evident in sporadic congenital cataract cases, which constitute the majority of patients and pose significant difficulties in identifying an underlying genetic etiology (Wirth et al., 2002). Previous studies have highlighted challenges in the early diagnosis and prognostic assessment of congenital cataracts. Early recognition is critical for timely intervention and improved treatment outcomes (Duret et al., 2019). These findings underscore the need for early diagnosis and prompt clinical intervention to prevent blindness or severe vision loss. Accordingly, NGS results have substantially reshaped genetic counseling for both parents and affected individuals. Therefore, NGS testing is recommended for affected children and for patients themselves, as it supports informed decisions and more precise clinical management (Li et al., 2018).

Most cases of congenital cataracts are sporadic, and only about 18.0% of patients report a family history (Wirth et al., 2002). In our cohort of sporadic pediatric cataracts, half of the detected variants were likely due to *de novo* heterozygous mutations in autosomal dominant genes (3/6). One-third were X-linked variants (2/6). One case carried a compound heterozygous mutation in an autosomal recessive gene (1/6). Overall, three variants were *de novo*. Notably, the two probands with X-linked syndromic cataract in our study were both conceived by *in vitro* fertilization (IVF).

Sporadic case #3 harbored a novel *BCOR* variant NM_001123385:c.4862del, p. (Pro1621Argfs*53). This variant was associated with oculo-facio-cardio-dental (OFCD) syndrome. OFCD syndrome is a rare congenital disorder. It is characterized by ocular, facial, cardiac, and dental anomalies. It was first described by Hayward et al. (1980), Gorlin et al. (1996). Because OFCD is characterized by multisystem malformations and marked phenotypic variability, establishing a definitive diagnosis can be challenging. This is especially true in patients with atypical features. In sporadic case #3, the proband had bilateral congenital cataract. The most evident additional findings were delayed eruption of deciduous teeth and oligodontia. In most reported OFCD cases, facial, oral, and limb abnormalities are mild in childhood. However, these features become more apparent during adolescence. Moreover, the later development of secondary glaucoma indicates the need for regular follow-up in patients with OFCD. Such monitoring may help prevent this complication (Zhang J. et al., 2019). In sporadic case #4, we identified a *de novo* X-linked missense variant in *OCRL* (OMIM 300535), NM_000276.4:c.1426A>G, p. (Thr476Ala). This variant is classified as VUS by ACGS and as LP by ACMG. This variant is located in the 5-phosphatase domain and is associated with Lowe syndrome. Lowe syndrome is a multisystem disorder. It is characterized by ocular abnormalities, neurological involvement, and Fanconi-type renal dysfunction (Charnas et al., 1991; Sharma et al., 2015). The causative gene, *OCRL*, was identified on the X chromosome in 1992. *OCRL* encodes a 5-phosphatase (Conduit et al., 2012). This enzyme preferentially acts on phosphatidylinositol 4,5-bisphosphate (PI(4,5)P2) (Attree et al., 1992). To date, most reported genetic defects in Lowe syndrome and Dent disease are deletions, frameshift variants, or nonsense (stop-gain) mutations. In contrast, splicing variants and missense mutations account for a smaller proportion (Hichri et al., 2011). Most missense variants cluster in the 5-phosphatase domain. The synaptojanin crystal structure was first used as a structural template for analysis (Pirruccello and De Camilli, 2012). This study reported that most missense variants in patients with Lowe syndrome involve conserved residues in 5-phosphatases. These changes can directly impair protein folding, substrate binding, or catalytic activity (Tsujishita et al., 2001). Patients with Lowe syndrome have an estimated life expectancy of approximately 40 years. Mortality is most often related to chronic kidney disease (CKD) and its complications (Zaniew et al., 2018). For children with congenital cataracts and systemic abnormalities, our findings underscore the value of early genetic diagnosis. Identification of the causative gene can support earlier risk assessment, targeted prevention, and timely management. Notably, both sporadic cases of X-linked syndromic cataract in this cohort involved children conceived by *in vitro* fertilization (IVF). Their dizygotic twin siblings showed no corresponding variants by Sanger sequencing. Given the small number of reported cases and the lack of mechanistic evidence, we could not determine whether IVF influences the risk of sex-chromosome variants compared with natural conception. A further limitation is that parentage (kinship) testing was not performed for the parents of these two probands.

Variants in the paired box gene 6 (*PAX6*) on chromosome 11p13 are the most common cause of congenital aniridia, a rare disorder affecting the development of multiple ocular structures. In this study, using an NGS-based approach, we identified novel PAX6 variants in two familial cases of congenital cataract and a *PAX6* fragment deletion in one sporadic case. These findings demonstrate the power of NGS for molecular diagnosis in congenital cataracts, particularly in cases where clinical phenotyping is incomplete. By providing a precise genetic diagnosis, NGS helps overcome a key challenge in genetic counseling for affected families—accurate assessment of inheritance risk (Lee et al., 2008; Beby et al., 2011; Luo et al., 2012). Beyond iris hypoplasia, patients may also present with other congenital ocular defects. These include cataracts, foveal hypoplasia, nystagmus, corneal opacity, lens dislocation, and glaucoma. These abnormalities can lead to substantial vision loss (Khan and Aldahmesh, 2008; Chien et al., 2009; Zhang et al., 2009; Aggarwal et al., 2011). Studies based on the haploinsufficiency model suggest that splicing and frameshift variants can produce truncated proteins. Variants of this type, including those described above, are likely to act through nonsense-mediated mRNA decay (NMD). In NMD, the mutant transcript is degraded, which reduces protein translation (Figure 5d) (Neethirajan et al., 2004; Tzoulaki et al., 2005). Our results expand the variant spectrum of PAX6 and further strengthen the genetic basis of aniridia. The newly identified variants improve the accuracy of variant interpretation. They also support more robust genotype–phenotype correlation analyses. Together, these findings provide a stronger foundation for genetic counseling and prenatal diagnosis in families affected by aniridia.

Previous studies have reported that targeted gene panel sequencing and WES improve the mutation detection rate (Zhai et al., 2017; Li et al., 2016; Ma et al., 2016). Identifying pathogenic variants can advance our understanding of crystalline lens function and the pathophysiology of congenital cataract. Consistent with these reports, we detected potentially pathogenic variants in 12 genes in our cohort. These genes comprise 105 exons and span 39.91 kb of genomic DNA.

This study has several limitations. First, we used WES rather than WGS. Therefore, deep intronic variants, distal regulatory variants, and complex structural variants may have been missed. Second, due to limited funding, we included only 40 cases with bilateral congenital cataract, and we did not perform WES in unilateral pediatric cataract cases diagnosed during the same period. Third, we could not conduct segregation analyses in additional unaffected family members. Fourth, some participants had undergone cataract surgery before enrollment. Thus, phenotypic data were obtained mainly from medical records or intraoperative video recordings. Fifth, seven variants were classified as variants of uncertain significance (VUS) under ACGS criteria, including four familial and three sporadic cases, and their pathogenicity requires further confirmation. Finally, functional assays were not performed, which may affect variant classification. Despite these limitations, our results support the clinical utility of WES for clarifying the genetic basis of congenital cataract. This work expands the variant spectrum of cataract-associated genes and strengthens genetic counseling. Future studies integrating WGS, transcriptomics, and functional assays may further increase diagnostic yield and deepen our understanding of disease mechanisms.

## Conclusion

In conclusion, this study demonstrates the clinical utility of WES for the genetic diagnosis of congenital cataract. We assessed the clinical features and molecular genetic findings in 40 Chinese probands with congenital cataract. We identified 15 putative pathogenic variants, including seven novel variants and eight recurrent variants. These results expand the variant spectrum of congenital cataract and broaden the associated phenotypic range. This information may support molecular diagnosis and precision care. Identifying cataract-associated causal variants also improves our understanding of lens biology and cataract pathogenesis. Further studies are needed to define the functional consequences of these variants and to confirm their pathogenicity.

## Data Availability

The datasets presented in this study can be found in online repositories. The names of the repository/repositories and accession number(s) can be found at: https://www.ncbi.nlm.nih.gov/, PRJNA1097220.

## Supplementary material

SUPPLEMENTARY FIGURE S1 The prediction of the tertiary structure of variants. a: CRYBB3 wild-type and missense mutant amino acid at position 156(p.Gly156Arg). b: CRYBB2 wild-type and missense mutant amino acid at position 188(p.Arg188Cys). c: CRYGS wild-type and missense mutant amino acid at position 83(p.Cys83Tyr). d: CRYAA wild-type and missense mutant amino acid at position 12(p.Arg12Cys). e: GJA8 wild-type and missense mutant amino acid at position 44(p.Val44Ala). f: GJA8 wild-type and missense mutant amino acid at position 45(p.Trp45Arg). g: OCRL wild-type and missense mutant amino acid at position 476 (p.Thr476Ala).

SUPPLEMENTARY FIGURE S2 Pedigrees of the families with variant of unknown significance (VUS).

SUPPLEMENTARY FIGURE S3 Pedigrees of families without identified pathogenic/likely pathogenic variants.
