# Supplementary material for: Genetic analysis and clinical characteristics of sporadic and familial congenital cataracts in southern Chinese families
Source: Front Genet. 2026 Feb 26;17:1744173. doi: 10.3389/fgene.2026.1744173 (PMC12978871; doi:10.3389/fgene.2026.1744173)
Supplement: Supplementary file 3 [file Table1.docx]

**Supp. Table S1. Clinical features in 40 families with sporadic and familial cataracts**

| **Family(**  **Proband)** | **Age of onset** | **Cataract**  **description** | **Procedures/age** | **IOP** | **HCD/age** | **AXL/age** | **BCVA(logMAR)** | **Additional features**  **(Extraocular features)** |
| --- | --- | --- | --- | --- | --- | --- | --- | --- |
| F#1 | SB | Lamellar cataract | BL Lensectomy+IOL/25y | R15.1 L17.1(PTS)  R22.5 L25.0(PO 7d) | R9.8  L？/25y | R22.31  L21.66/25y | R2.1 L0.5  (PO 1d) | BL Nystagmus Sensory exotropia Posterior staphyloma |
| F#2 | 6y | N/K | BL Lensectomy/6y  IOL/11y  R dislocated IOL/24y | R17.0 L19.0(PTS) | R ?  L10.7/24y | R25.04 L25.00/24y | R1.1 L1.2  (PO 14y) | BL Nystagmus Amblyopia |
| F#3 | 3y | Embryonic nuclear  cataract | BL Lensectomy  +IOL/3y | N/K | R11.6  L11.7/3y | R24.76 L23.69/3y | R1.6 L0.7(PST)  R0.8 L0.3(PO 3m) | BL Trichiasis |
| F#4 | 5y | Embryonic nuclear  cataract | Nil | R15.2 L15.4 | R10.7  L10.9/10y | R21.57 L21.56/10y | R0.1 L0.2 | BL Intermittent exotropia |
| F#5 | 2y | Lamellar cataract | BL Lensectomy  +IOL/2y | R15.4 L15.5(PTS)  R13.9 L12.2(PO 3m) | R11.9  L12.4/2y | R20.6 L20.74/2y | R0.9 L0.8(PST) | Nil |
| F#6 | SB | Lamellar cataract | BL Lensectomy/1y | N/K | N/K | R20.08 L20.20/1y | N/K | BL Nystagmus |
| F#7 | 6y | Embryonic Nuclear  cataract | Nil | R13.8 L19.2 | N/K | N/K | R0.9 L0.8 | BL Aniridia Nystagmus  Foveal hypoplasia |
| F#8 | SB | Total | L Lensectomy+IOL/28y  R Lensectomy+IOL/42y | R9.5 L19.7(PTS)  R18.1 L13.9(PO 7d) | R11.1  L11.1/42y | R25.67  L24.60/42y | R1.2 L1.0(PST)  R1.1 L1.0(PO 7d) | BL Aniridia Nystagmus |
| F#9 | 6y | Coralliform cataract | Nil | R22.0 L21.0 | R12.2  L12.1/11y | R22.63  L22.76/11y | R0.2 L0.2 | BL Concomitant esotropIa |
| S#1 | 6y | Embryonic nuclear  cataract | L Lensectomy+IOL/6y | R12.9 L15.4(PTS)  R13.9 L12.0(PO 3m) | R11.4  L11.3/7y | R20.92  L20.06/7y | R0.1 L0.6(PST)  R0 L0.3 (PO 7d) | L Amblyopia |
| S#2 | 5m | N/K | BL Lensectomy/5m  BL IOL/5y | R17.1 L29.2(PTS)  R12.5 L18.9(PO 11m) | N/K | R25.36  L25.92/5y | R1.3 L1.3(PST)  R0.6 L0.7(PO 11m) | BL EsotropIa Nystagmus  Posterior staphyloma |
| **Family(**  **Proband)** | **Age of onset** | **Cataract**  **description** | **Procedures/age** | **IOP** | **HCD** | **AXL** | **BCVA** | **Additional features**  **(Extraocular features)** |
| S#3 | 3m | Posterior polar  cataract | BL Lensectomy/7m | N/K | N/K | N/K | N/K | BL Lower eyelid inversion  Patent foramen ovale  Dental dysmorphologies and delayed eruption  Broad nasal tip (OFCD syndrome) |
| S#4 | 1y | Total cataract | BL Lensectomy+IOL/2y | R17.0 L16.0(PTS)  R19.0 L14.0(PO 7d) | N/K | R21.41  L21.59/2y | R0.5 L0.5(PO 1d) | Proteinuria Hematuria  Micropenis Growth retardation  （Lowe syndrome） |
| S#5 | 3y | Embryonic nuclear  cataract | BL Lensectomy+IOL/4y | R13.0 L16.0(PO 1d) | R11.5  L11.0/4y | R21.47  L20.96/4y | R0.9 L0.9(PO 6m) | BL Aniridia Nystagmus  Foveal hypoplasia |
| S#6 | 1y | R Posterior subcapsular cataract L Total | BL Lensectomy+IOL/2y | R20.7 L16.6(PTS)  R16.6 L13.2(PO 7m) | R11.7  L10.8/3y | R20.76  L21.12/3y | R0.8 L3.0(PST)  R0.4 L0.6(PO 7m) | Nil |
| F#10 | 6y | Embryonic nuclear and  cortex cataract | BL Lensectomy+IOL/6y | R10.9 L10.0(PTS)  R13.3 L12.7(PO 1d) | R？  L11.2/12y | R22.71  L22.47/12y | R0.5 L0.5(PST)  R0.2 L0.3(PO 7d) | BL EsotropIa Nystagmus |
| F#11 | 5y | Embryonic nuclear  pulverulent cataract | Nil | R18.8 L25.9 | N/K | N/K | R0.2 L0.1 | Nil |
| F#12 | SB | Embryonic nuclear  cataract | BL Lensectomy+IOL/11y | R14.9 L17.0(PTS)  R11.1 L11.9(PO 3m) | R12.2  L12.7/11y | R22.78  L22.77/11y | R0.3 L0.3(PST)  R0.1 L0.1(PO 3m) | Nil |
| F#13 | 5y | Total | BL Lensectomy+IOL/6y | R23.3 L25.0(PTS)  R15.1 L13.6(PO 1m) | R11.8  L?/6y | R21.93  L21.90/6y | R0.4 L0.4(PO 1m)  R0.1 L0.1(PO 6m) | Nil |
| F#14 | 6m | Posterior polar  cataract | BL Lensectomy+IOL/6y | R18.0 L18.5(PTS)  R13.9 L13.0(PO 4m) | R11.2  L10.9/6y | R22.92  L22.76/6y | R0.5 L0.7(PST)  R0.3 L0.2(PO 4m) | Nil |
| F#15 | 48y | Coralliform cataract | R Lensectomy+IOL/50y | R10.8 L11.6(PTS)  R10.6 L10.6(PO 1m) | R11.8  L11.4/50y | R22.53  L22.52/50y | R0.3 L0.1(PST)  R0.1 L0(PO 1m) | Nil |
| **Family(**  **Proband)** | **Age of onset** | **Cataract**  **description** | **Procedures/age** | **IOP** | **HCD** | **AXL** | **BCVA** | **Additional features**  **(Extraocular features)** |
| F#16 | 4y | Embryonic nuclear  pulverulent cataract | BL Lensectomy+IOL/5y | R15.7 L19.1(PTS)  R14.9 L10.3(PO 3m) | R12.5  L11.8/5y | R23.50  L23.69/5y | R0.3 L0.5(PST)  R0.1 L0.2(PO 3m) | Nil |
| F#17 | 1y | Posterior polar  cataract | BL Lensectomy+IOL/2y | N/K | N/K | R21.74  L21.54/2y | ou 0.4(PO 3m) | Nil |
| F#18 | 4y | R Posterior polar  cataract L Total | BL Lensectomy+IOL/6y | R18.4 L10.9(PTS)  R14.6 L20.2(PO 1d) | R11.8  L?/6y | R21.76  L21.95/6y | R0.8 L1.0(PST)  R0.3 L0.3(PO 9d) | Nil |
| F#19 | 4y | pulverulent cataract | BL Lensectomy+IOL/5y | R16.5 L9.9(PTS)  R16.1 L15.7(PO 1m) | R11.8  L11.9/5y | R24.27  L23.90/5y | R0.5 L0.4(PST)  R0.1 L0.1(PO 3m) | Nil |
| S#7 | 6y | Punctate in lens Cortex cataract | BL Lensectomy+IOL/27y | R16.4 L17.9(PTS)  R13.8 L14.7(PO 37d) | R12.0  L11.9/27y | R29.83  L30.01/27y | R0.3 L0.2(PST)  R0.1 L0.1(PO 37d) | Nil |
| S#8 | 6m | N/K | L Lensectomy+IOL/7y | R17.2 L17.8(PTS)  R17.6 L14.4(PO 1d) | R11.51  L11.19/7y | R22.9  L21.69/7y | R0.2 L0.6(PST)  R0.2 L0.2(PO 7d) | Kawasaki disease |
| S#9 | SB | R Total  L Pupillary atresia | Nil | N/K | N/K | N/K | R LP L NLP | BL Iridectopia Nystagmus Ptosis Wide interocular distance  Broad nasal bridge  Dental dysmorphologies  Mandibular prognathism  Polycystic kidney Hydrocephalus |
| S#10 | 8y | Total | BL Lensectomy+IOL/9y | R10.4 L13.7(PTS)  R16.5 L14.9(PO 7d) | R10.7  L10.8/9y | R21.79  L21.45/9y | R0.5 L0.4(PST)  R0.1 L0.1(PO 7d) | Mental retardation |
| S#11 | 5y | Posterior polar  cataract | R Lensectomy+IOL/6y | R19.7 L17.6(PTS)  R15.0 L16.4(PO 1y) | R11.81  L11.71/6y | R23.06  L21.90/9y | R1.2 L0.2(PST)  R0.4 L0.3(PO 1y) | BL Amblyopia |
| S#12 | SB | N/K | BL Lensectomy/1y | R24.2 L24.0 | N/K | N/K | R0.7 L0.7 | Cricoid cartilage softening  Mandibular hypoplasia  Dental dysmorphologies and Delayed eruption  Delayed growth and development |
| **Family(**  **Proband)** | **Age of onset** | **Cataract**  **description** | **Procedures/age** | **IOP** | **HCD** | **AXL** | **BCVA** | **Additional features**  **(Extraocular features)** |
| S#13 | 2m | Embryonic nuclear  cataract | BL Lensectomy/8m | R25.0 L17.0(PO 22d) | N/K | R19.95  L19.72/8m | N/K | Nil |
| S#14 | SB | Embryonic nuclear  cataract | BL Lensectomy/6m  L IOL/2y | R20.0 L20.0(PO 1y) | N/K | R21.00  L20.34/2y | N/K | BL Nystagmus |
| S#15 | 2y | pulverulent cataract | BL Lensectomy+IOL/6y | N/K | R11.4  L11.4/6y | R23.52  L23.52/6y | R0.7 L1.2(PST)  R0.2 L0.3(PO 6m) | Nil |
| S#16 | 11y | Embryonic nuclear Y-sutural | Nil | R19.3 L17.2 | N/K | N/K | R0 L0 | Nil |
| S#17 | 1y | Embryonic nuclear  cataract | BL Lensectomy+IOL/2y | R15.8 L10.6(PTS)  R10.4 L?(PO 1m) | N/K | R20.50  L20.00/2y | R0.5 L0.5(PO 6m) | Nil |
| S#18 | 18y | Coralliform cataract | Nil | R13.3 L12.3 | N/K | N/K | R0 L0 | Nil |
| S#19 | 2y | Total | BL Lensectomy+IOL/2y | R9.0 L9.0(PO 7d) | R?  L11.2/2y | R22.46  L21.96/2y | OU0.6(PO 3m) | BL Nystagmus |
| S#20 | 4y | Embryonic nuclear  cataract | L Lensectomy+IOL/24y | R13.8 L14.0(PTS)  R11.9 L13.8(PO 3m) | R11.2  L11.2/24y | R24.62  L27.84/24y | R2.1 L2.4(PST)  R0.3 L2.4(PO 3m) | BL Exotropia |
| S#21 | 4y | Central cortical cataract | R Lensectomy+IOL/9y | R16.1 L17.4(PTS)  R13.8 L10.9(PO 18m) | R12.6  L12.5/9y | R25.57  L25.27/9y | R0.4 L0.4(PST)  R0.1 L0.2(PO 18m) | BL Irregular astigmatism |

**F Familial, S Sporadic, L Left, R Right, SB Since birth, BL bilateral, PC Posterior chamber, IOL Intraocular Lens, IOP Intraocular pressure, HCD Horizontal Corneal Diameter in mm and age of measurement, AXL Axial Length in mm and age of measurement, BCVA best corrected visual acuity, N/K Not known, y year, m month, d day. PTS Prior to surgery, PO Postoperation.**
