# Supplementary figures and images for: Genetic analysis and clinical characteristics of sporadic and familial congenital cataracts in southern Chinese families
Source: Front Genet. 2026 Feb 26;17:1744173. doi: 10.3389/fgene.2026.1744173 (PMC12978871; doi:10.3389/fgene.2026.1744173)

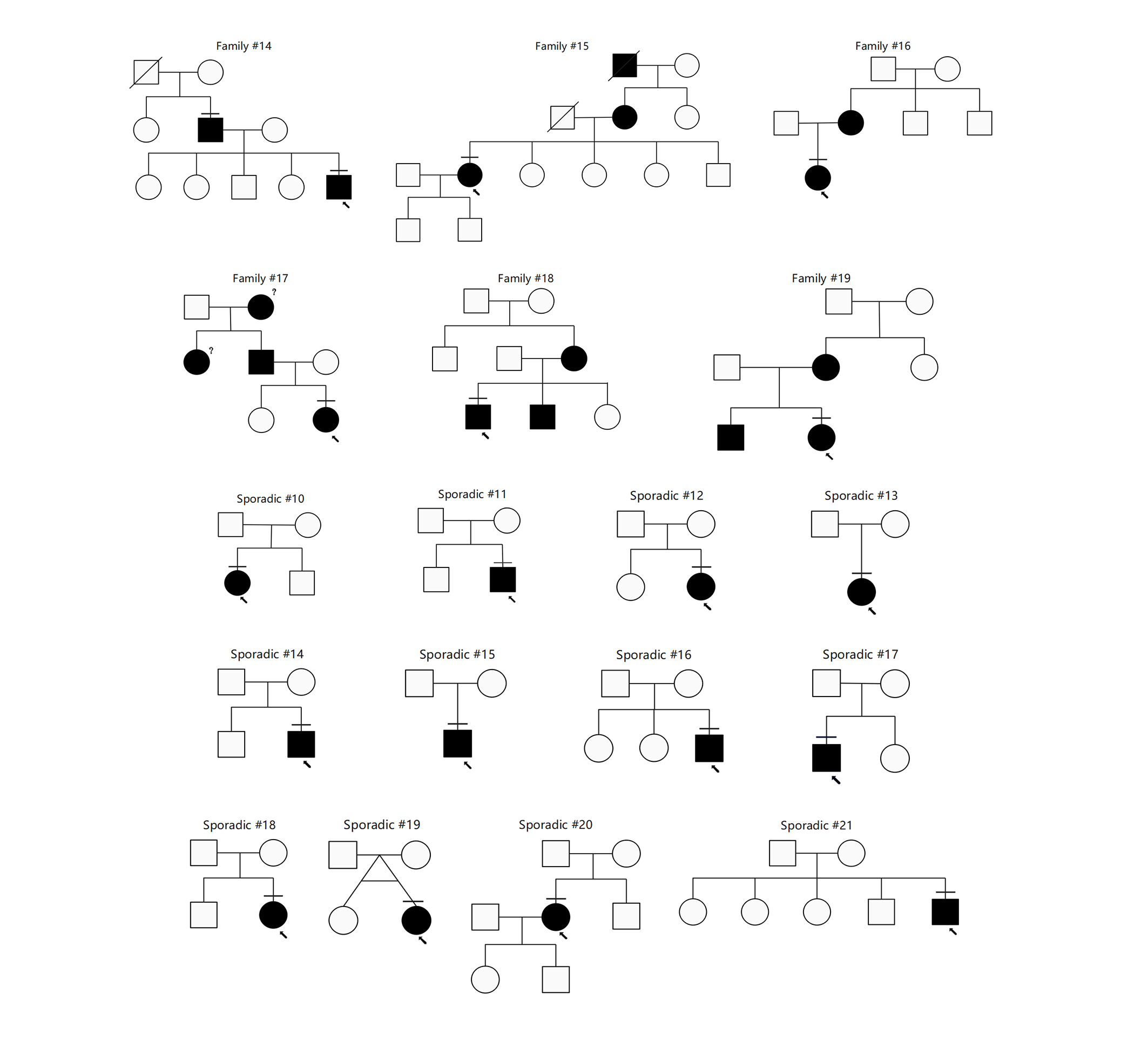

Supplement: Supplementary file 4 [file Image3.tif]

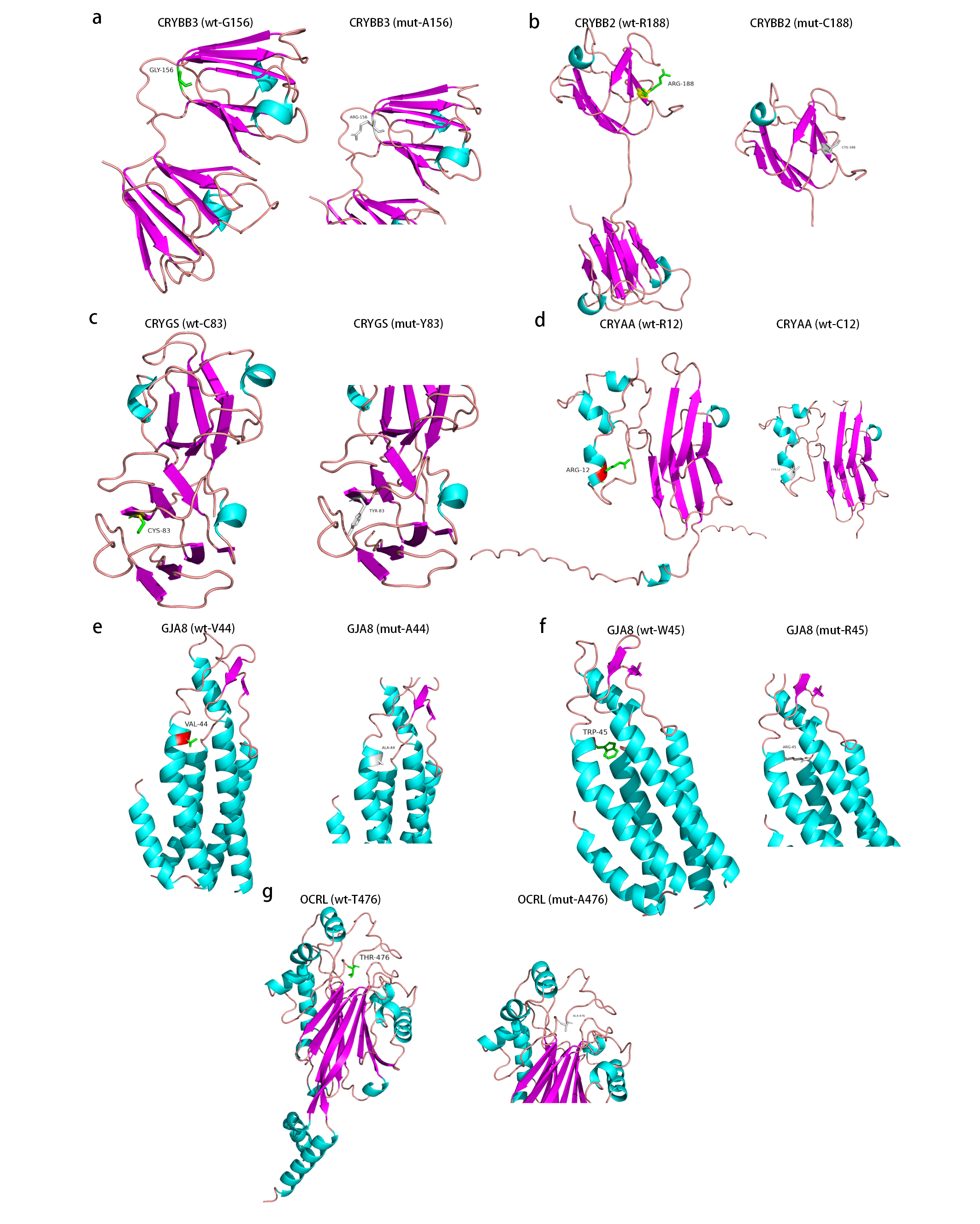

Supplement: Supplementary file 5 [file Image1.tif]

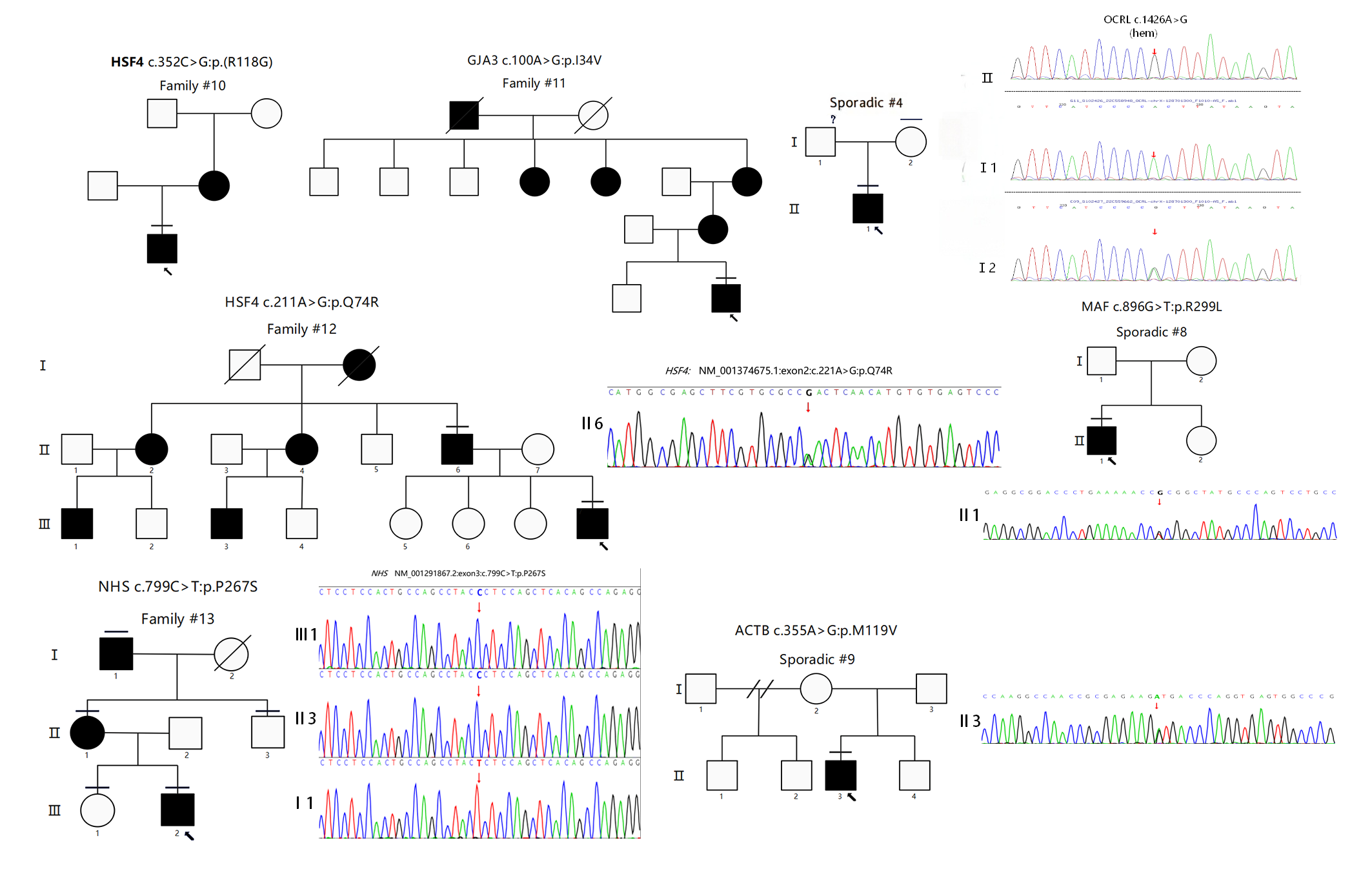

Supplement: Supplementary file 6 [file Image2.png]
