# Supplementary material for: Genetic analysis and clinical characteristics of sporadic and familial congenital cataracts in southern Chinese families
Source: Front Genet. 2026 Feb 26;17:1744173. doi: 10.3389/fgene.2026.1744173 (PMC12978871; doi:10.3389/fgene.2026.1744173)
Supplement: Supplementary file 7 [file Table4.docx]

1.Whole Exome Sequencing (WES)

Regarding the WES testing, there are no gaps or limited coverage analysis in the exon regions of the known genes related to hereditary cataract. Moreover, comprehensive analyses will be carried out for the variants included in the HGMD/Clinvar database as well as those predicted by the Splice AI software to affect splicing.

2.CAT-MAP hereditary cataract database

CatMap was compiled as a curated reference database and gene map for inherited and agerelated forms of cataract (https://cat-map.wustl.edu/, accessed on 12 June 2024). Keyword searches of PubMed (https://pubmed.ncbi.nlm.nih.gov/, accessed on 12 June 2024), Online Mendelian Inheritance in Man (OMIM, https://omim.org/, accessed on 12 June 2024), and other National Center for Biotechnology Information (NCBI, https: //www.ncbi.nlm.nih.gov/, accessed on 12 June 2024) databases are used to identify relevant peer-reviewed literature reporting loci, genes, mutations/variants, mode-of-inheritance, geographic origin, cataract appearance, along with any co-existing ocular and/or systemic phenotypes. Genes and loci are listed in human chromosome order with genomic coordinates (Genome Reference Consortium Human Build 38/GRCh38) and syntenic mouse and other animal genes and models for cataract are appended. Mutations and variants are numbered using standard nomenclature recommendations starting with the A of the translation start-codon (A1TG) and/or with reference sequence (rs) numbers.
